# The Dry Earth System

**Published:** 1875-07

**Authors:** 


### THE DRY EARTH SYSTEM.

We have uttered numerous warnings against the very common use of deep vaults in towns not supplied with a complete system of sewers. Beyond a doubt this practice has occasioned much disease and many deaths. The chief cause of typhoid is the water supply poisoned by proximity to receptacles for human excreta. A prolific source of disease is found in the noxious exhalations from vaults into which the purifying influences of sun and air never enter.

It is infinitely desirable to get rid of these dangerous and offensive influences, and it is unquestionably possible to escape them. The remedy is a privy without a vault, and the use of earth as a deodorizer. The edifice costs no more and the expense of the vault is saved. The trouble of attending it is greater, but the difference is trifling when compared with the advantages gained.

The building is the usual structure in a place which is dry rather than wet. It may be within ten feet of the back door, and never be detected, as it gives off no odor. Its back as high as the seat, must be hinged, so that it can be swung up and the accumulations removed from time to time. Of greater importance is it that the entire seat be hinged, so that the accumulations may be stirred up with a hoe frequently, and mixed with the earth which has been sprinkled in. Dry earth must be kept always at hand, and a small shovel or scoop should be conveniently placed, so that each occupant shall understand that the excreta is to be covered at once. Fluids must never be thrown in, as they demand a much greater supply of earth for their absorption than the solid excreta. The slops from sleeping apartments are readily disposed of by pouring them upon loose earth exposed to the sun's rays, and immediately working the earth over with a hoe or rake. The land is enriched and the fluids are disposed of without offence.

As will be seen, the chief things in adapting the ordinary privy to the earth-system is to have plenty of room to agitate the mass, and plenty of earth to apply. By hinging a door in the rear, and by also hinging the entire seat so that they can be lifted up, thus giving ample access to the space beneath the seat; and by devoting ten minutes each week to the simple and unobjectionable work of mixing the contents, so that each particle of excrement may be brought in contact with a particle of earth—nothing offensive can emenate from the place.

When the space between the seat and the ground gets nearly full the contents must be removed and kept for use as a fertilizer. It is about as valuable as guano, and should be preserved in barrels or under cover so that the rains may not dissolve and disperse its fertilizing properties. There is absolutely no system as perfect as the dry earth system, properly employed, and it is wholly impossible to employ it effectively in any under-ground vault. The dryer the receptacle is, the more perfect is the application of the system. Vaults are abominations, are quite unnecessary, and should be prohibited by law, for they have killed their thousands and tens of thousands.

---

SMALL-POX DEFORMITIES. — We know of no surer preventive of "pitting" in small-pox than the application of pure honey, right from the comb. We paint the ulcers over, not less than three times every twenty-four hours, and the skin comes out as smooth and free from depressions as that of a child. Honey, so applied, is a remedy for most cracks and irritations and fissures of the skin.

---

CARRYING CANES.—Carry a cane, if you will, but don't carry it under your arm with the end projecting horizontally. Even the most amiable person will resent an attack in the face with the dirty extremity of a careless fellow's cane. A real gentleman will be sure not to infringe upon the rights of others, especially in the crowded streets and other public places. Besides the practice is a dangerous one to the eyes of the people in the rear.

---

Love reckons hours for months, and days for years, and every little absence is an age.

---

Nothing is too high for the daring of mortals. We storm Heaven itself in our folly.

---

Whole years of joy glide unperceived away, while sorrow counts the minutes as they pass.

